# Toward FGF2 reduction in cultured meat media: polyphenol salts enhance growth and differentiation of bESC aggregates

**DOI:** 10.3389/fnut.2025.1669909

**Published:** 2025-12-03

**Authors:** Gaya Savyon, Ruchama Korol, Muhammad Abdel-Haq, Sara Ben Saadon, Dana Bistritz, Hodaya Lankry, Yuval Peled, Roni Rak, Berta Levavi-Sivan, Ofra Benny, Abraham J. Domb, Iftach Nachman

**Affiliations:** 1 School of Neurobiology, Biochemistry and Biophysics, Tel Aviv University, Tel Aviv, Israel; 2 School of Pharmacy-Faculty of Medicine, The Hebrew University of Jerusalem, Jerusalem, Israel; 3 Department of Animal Sciences, The Robert H. Smith Faculty of Agriculture, Food, and Environment, Hebrew University of Jerusalem, Rehovot, Israel; 4 Institute for Animal Sciences, Agricultural Research Organization, Volcani Institute, Rishon LeZion, Israel

**Keywords:** low cost media, bovine embryonic stem cells, cell aggregate, polyphenols, serum-free media

## Abstract

Recombinant growth factors, particularly fibroblast growth factor 2 (FGF2), are major cost drivers in the production of cultured meat. In this study, we investigated the potential of polyphenol salts to reduce reliance on FGF2 in media supporting the proliferation and mesodermal differentiation of bovine embryonic stem cell (bESC) aggregates. The activation potential of these salts was first verified using a luciferase reporter assay in COS-7 cells expressing human FGFR1. Several compounds, particularly Na-Quercetin, induced strong, dose-dependent FGFR1 activation with sub-nanomolar EC₅₀ values, comparable to FGF2. We then evaluated the use of three of the salts Sodium-Curcumin (NaCur), Potassium-Naringenin (K-Ng) and Sodium-Quercetin (Na-Q) on bESC aggregates. NaCur significantly enhanced aggregate growth under reduced FGF2 conditions, restoring proliferation to levels exceeding those observed with 20 ng/mL FGF2 alone. Additionally, NaCur supported mesodermal differentiation, as indicated by Brachyury expression, when combined with low-dose FGF2. K-Ng and Na-Q improved aggregate growth in the absence of FGF2 serum-free conditions but were insufficient to support mesodermal differentiation. These findings suggest that NaCur can reduce the required concentration of recombinant FGF2 while supporting both proliferation and differentiation, whereas K-Ng and Na-Q may be better suited for the early expansion phase. Our results highlight the potential of using polyphenol supplementation as a strategy to lower medium costs in cultured meat production systems.

## Introduction

The global demand for sustainable and high-quality protein sources is rapidly increasing, driven by population growth and growing awareness of the environmental impacts of conventional animal agriculture. Cultured meat (CM), produced directly from animal cells without the need for livestock farming, has been proposed as a potential alternative to traditional meat production systems (1). Recent life cycle assessments (LCAs) indicate that CM could reduce greenhouse gas emissions, land use, and biodiversity loss compared to conventional beef production. However, these projections remain highly model-dependent and subject to ongoing debate. In particular, comprehensive assessments that also consider the ecosystem services and socio-economic contributions of livestock are still lacking, and balanced perspectives emphasize that livestock can play an essential role in sustainable agro-ecosystems (2–4). Nevertheless, given the environmental pressures associated with current meat production systems and the projected growth in global protein demand, developing economically viable and resource-efficient CM technologies remains a key objective toward diversifying future protein supply chains.

One of the main barriers to achieving economic and environmental viability is the reliance on expensive, highly refined growth factors such as fibroblast growth factor 2 (FGF2), which contribute significantly to the carbon footprint and costs of CM production (5, 6). Although used in minute quantities, growth factors contribute disproportionately to the environmental footprint of cell culture media, with recent life cycle assessments estimating that the production of just 1 mg of FGF2 can emit up to 0.04 kg of CO₂ equivalents (6).

FGF2 plays a pivotal role in the *in vitro* expansion of embryonic stem cells (ESCs), supporting their proliferation and maintaining their pluripotent state. FGF2 activates key signaling pathways such as MAPK/ERK and PI3K/AKT, which are essential for sustaining cell viability and promoting rapid proliferation (7). Beyond its role in self-renewal, FGF2 is also critically involved in the early stages of mesodermal differentiation, facilitating the transition of pluripotent cells toward mesodermal lineages by modulating the expression of transcription factors such as Brachyury (8). This dual function of FGF2 in supporting both proliferation and lineage specification makes it a central component of current growth and differentiation protocols aimed at producing muscle progenitors for CM applications.

Polyphenol molecules (<1,000 Da) were found to be able to activate particular signalling pathways that may lead to cell growth and differentiation (9). Unlike GFs, these small molecules are easier and less costly to manufacture or are readily available from bioresources and are biochemically more stable. There are more than 8,000 different types of polyphenols that have been identified so far. Many polyphenols were found to act as biological mediators with potential to substitute or enhance activity of cytokines (10). A comprehensive review on polyphenol molecules mimicking growth factors for tissue engineering has been recently published (11). This review focuses on the use of polyphenols as cell proliferation agents *in vivo* or in common tissue culture media that are rich with animal components such as serum. On the other hand, numerous publications report on the use of polyphenols as inhibitors of cell proliferation for use in cancer therapy (9, 12). Most polyphenols are water insoluble and thus their accessibility to cells in tissue culture is limited. Thus, water soluble polyphenols were prepared by converting them to phenolate salts by reacting them with NaOH or KOH in ethanol or in water (13).

In addition to the high cost of growth factors, the development of scalable culture systems presents a significant technological hurdle. Efficient cultivation of embryonic stem cells in suspension under shear forces remains a major challenge in the cultivated meat field, particularly for domestic species such as bovine. While recent years have seen significant progress in the derivation of stable bESC lines and in developing protocols for their differentiation into muscle lineage, most of these advances have relied on adherent culture systems (14, 15). To date, very few studies have demonstrated successful bESC proliferation and differentiation under suspension conditions, which are essential for translating stem cell cultivation into industrial-scale bioreactors (16).

Here we develop a three-dimensional culture system for bESC aggregates that supports both proliferation and mesodermal differentiation. We demonstrate that supplementing the culture medium with polyphenol salts can effectively reduce the reliance on FGF2 without compromising cell growth or differentiation capacity. This approach offers a scalable and economically viable solution, addressing two of the most critical bottlenecks in cultivated meat production.

## Results

**Figure 1:**
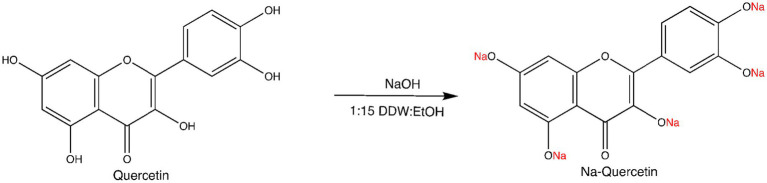
Synthesis of Sodium Quercetin salt. Quercetin was converted to its water-soluble sodium phenolate salt by reaction with NaOH in a 1:15 (v/v) mixture of double-distilled water (DDW) and ethanol. The reaction proceeds via deprotonation of phenolic hydroxyl groups and yields Na-Quercetin, confirmed by spectral analysis.

Water-soluble phenolate salts of Quercetin Curcumin and Naringin were prepared from the reaction of the, polyphenol with NaOH or KOH in ethanol or in water. The successful conversion of the parent polyphenols into their corresponding sodium and potassium salts was confirmed by Fourier-transform infrared (FTIR) spectroscopy and proton nuclear magnetic resonance (^1^H NMR). Characteristic FTIR peaks consistent with phenolate formation were observed in all salts, including the strong carbonyl (C=O) stretching band near 1,659 cm^−1^ and aromatic C=C stretching bands at 1604 cm^−1^ and 1,510 cm^−1^, which were retained from the parent structure. The disappearance of the broad O–H stretching band (typically 3,200–3,600 cm^−1^) confirmed deprotonation of the phenolic hydroxyl groups. Corresponding ^1^H NMR spectra showed upfield shifts of aromatic proton signals compared to the neutral compounds, further supporting formation of phenolate anions. Together, these spectroscopic features verify that the reaction proceeded via deprotonation of hydroxyl groups to yield the intended water-soluble sodium and potassium polyphenol salts. The compounds were prepared in high yield and confirmed by spectral analysis. The synthesis of Quercetin sodium salt is given in Figure 1.

In addition to being chemically straightforward, the synthesis of polyphenol salts is highly cost-effective. The reaction proceeds under ambient conditions using low-cost reagents such as sodium or potassium hydroxide, followed by filtration and drying steps (see cost estimates in Box 1).

In this study, we focused on FGF2 as a representative recombinant growth factor, demonstrating that polyphenol salts can either fully replace or partially reduce its required concentration in suspension cultures of bESCs. FGF2 was chosen because of its high cost, its short half-life under culture conditions, and its broad usage across diverse cell types and organisms relevant to cultivated meat production. Notably, FGF2 is required in multiple stages of the production process, including both proliferation and, in most cases, myogenic differentiation, making it a strategic target for substitution when aiming to reduce medium costs.

BOX 1 **Cost estimates of FGF2 and polyphenol salts for cultivated meat applications.**

FGF2 production cost estimates: The market price of FGF2 varies widely depending on three main factors: purity level, production scale, and regulatory classification (e.g., research-grade vs. food-grade). Catalog prices range from approximately $795 to $30,960 per milligram. Assuming a 1,000% markup, which is common in the biotechnology sector, this implies estimated production costs of: $72.27 to $2,814.55 per 1 mg FGF2.

To estimate the cost contribution of FGF2 to cultivated meat production, consider a low concentration of 10 ng/mL and a media requirement of 10 liters per kilogram of meat. This results in: 0.1 mg FGF2 per kilogram of cultivated meat. Meaning FGF2 cost contribution is approximately $7.23–$281 per cultivated meat kilogram.

Polyphenol salts production cost estimates: Based on current market prices, the production cost of water-soluble polyphenol salts is estimated at approximately two hundred USD per kilogram, assuming a polyphenol powder cost of $150/kg and minimal added processing costs. Polyphenol salts are effective at nanomolar to tens-of-nanomolar concentrations. For calculation purposes, we conservatively assume a very high working concentration of 100 nM and a molecular weight of 500 Da. At 100 nM, the mass of polyphenol salt required per liter is: 5 × 10^−5^ g. At $200/kg production cost, the cost per medium is: 10^−5^ $/L Thus, in 10 liters of medium (for 1 kg of cultivated meat), the Polyphenol cost contribution is approximately 10^−4^ $/Kg CM. Even under the most conservative scenario of maximum polyphenol use and minimum FGF2 use, the cost contribution of polyphenol salts is at least 10,000-fold lower than that of recombinant FGF2.

### Quercetin, Naringenin and Curcumin salts enhance metabolic activity of mouse fibroblasts in the absence of FGF2

**Figure 2:**
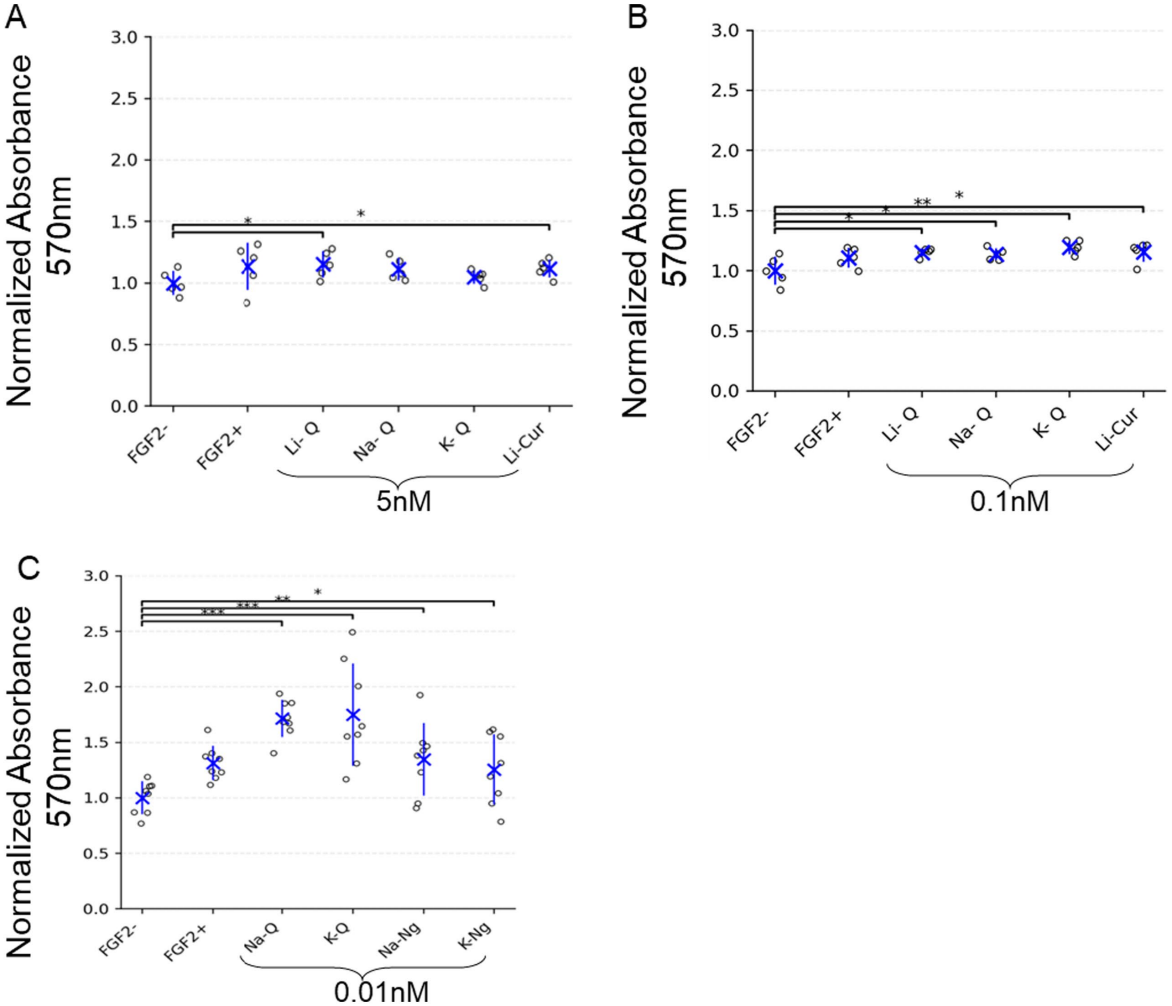
Polyphenol salts enhance metabolic activity of NIH/3 T3 cells in the absence of FGF2. **(A–C)** MTT assay results showing normalized absorbance at 570 nm (background-subtracted) for NIH/3 T3 cells cultured with either FGF2 (5 ng/mL) or various polyphenol salts at the indicated concentrations: **(A)** 5 nM, **(B)** 0.1 nM, and **(C)** 0.01 nM. Values are normalized to the negative control (FGF2-). Each dot represents an individual well, with blue crosses indicating group means and error bars representing mean ± SD. Significant differences are indicated with brackets and asterisks: *p < 0.05, **p < 0.01, ***p < 0.001. **(A)** *n* = 5 wells; **(B)** *n* = 5 wells; **(C)** *n* = 8 wells.

To evaluate whether polyphenol salts can promote cell growth and metabolic activity independently of fibroblast growth factor 2 (FGF2), we first tested their effects in a simple and well-established fibroblast model. NIH/3 T3 cells were cultured under low-serum conditions (1% FCS) with or without FGF2 supplementation and treated with increasing concentrations of the sodium, potassium, or lithium salts of Quercetin, Curcumin, or Naringenin. Metabolic activity was assessed by MTT assay after five days of culture. As expected, removal of FGF2 markedly reduced cell metabolic activity compared to the FGF2-treated control. All tested salts of Quercetin, Naringenin, and Curcumin improved metabolic activity under FGF2-free conditions. Interestingly, Quercetin showed the strongest enhancement at extremely low concentrations (0.01 nM). Naringenin also yielded a marked improvement in this sub-nanomolar range. In contrast, the positive effect of Lithium Curcumin (Li-Cur) was maintained but did not increase further when its concentration was reduced from 5 nM to 0.1 nM, therefore lower concentration of 0.01 nM was not tested. These findings suggest that higher concentrations of Quercetin may attenuate its beneficial effects, whereas Curcumin remains effective even at elevated doses (Figure 2).

### Low concentrations of Quercetin and Naringenin salts increase bMSC survival under low-serum conditions

**Figure 3:**
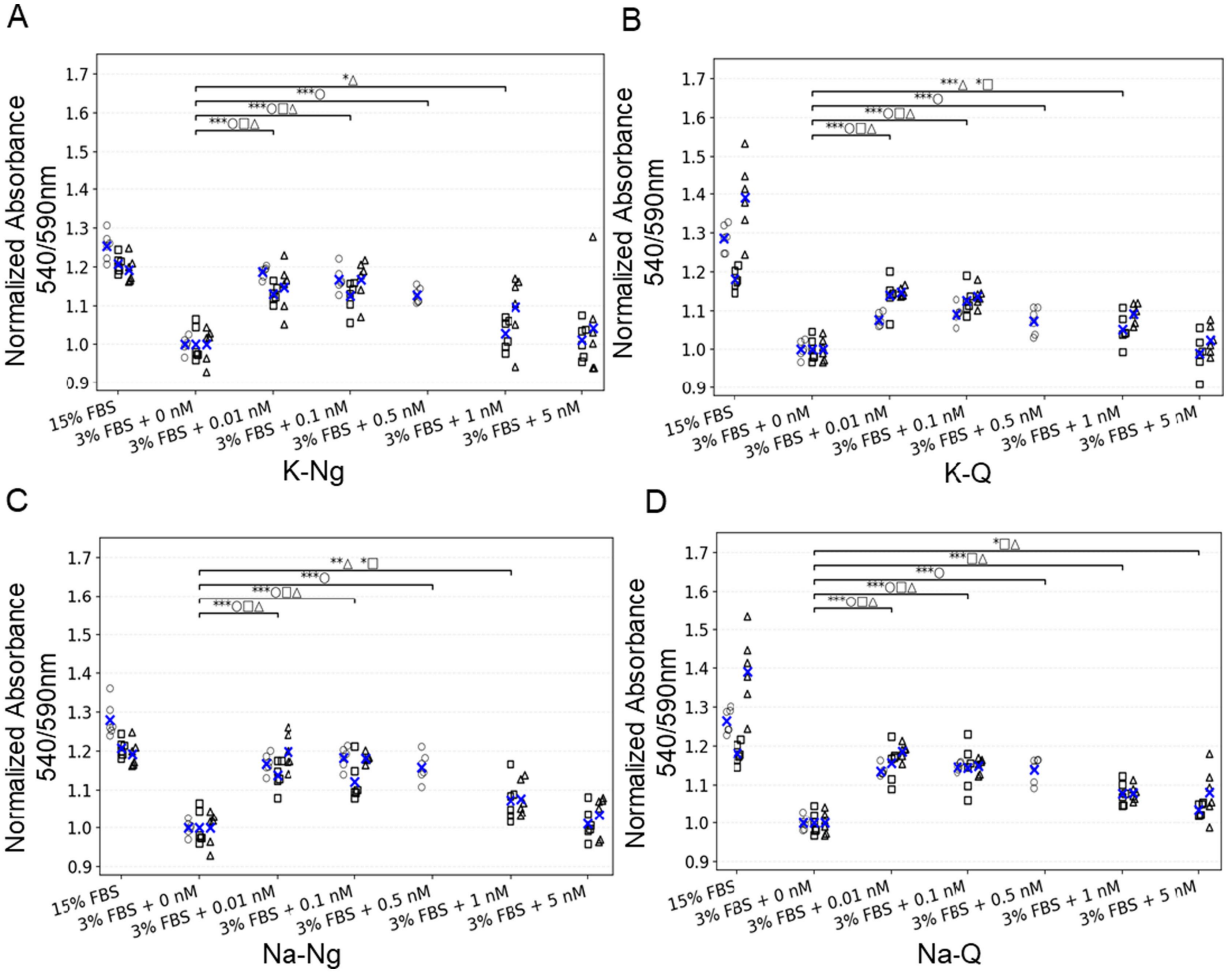
Low concentrations of polyphenol salts increase bMSC survival under low-serum conditions. **(A–D)** Normalized absorbance 540/590 nm for four polyphenols: **(A)** K-Ng, **(B)** K-Q, **(C)** Na-Ng, and **(D)** Na-Q. Values are normalized to the negative control (3% FBS + 0 nM). Each dot is an individual well plotted as an empty symbol for its replicate (○ rep a, □ rep b, △ rep c); blue × marks the mean of that replicate within each condition. Above the data, one bracket per comparison summarizes statistics versus the indicated control(s); bracket labels concatenate per-rep significance (e.g., ***□ **○ ***△). Asterisks denote one-sided *t*-tests (alternative: greater): *p* < 0.05 (), *p* < 0.01 (), *p* < 0.001 (). Brackets are shown only when at least one replicate is significant. Number of replicates per condition: 6.

We next moved to bovine mesenchymal stem cells (bMSCs), a more relevant cell line for cultivated meat. These cells are typically maintained in growth media containing 15% FBS, and reducing serum concentration is highly detrimental to their viability and proliferation. Supplementation with FGF2 was insufficient to restore normal growth under these low-serum conditions (Figure 3). To test whether polyphenol salts could mitigate the dependence on high serum, we examined the effects of Quercetin and Naringenin salts on bMSC survival and metabolic activity when FBS was reduced to 3% with the addition of insulin, transferrin, selenite, linoleic acid, and BSA (ITS-Plus). Cell metabolic activity, used as a proxy for viability and proliferation, was measured after five days of culture using the Alamar Blue colorimetric assay. Consistent with the 3 T3 results, both Quercetin and Naringenin salts markedly improved cell survival under serum-reduced conditions. The strongest effects were observed at sub-nanomolar concentrations (0.01 nM), indicating that these compounds can partially substitute for serum-derived growth support and sustain bMSC viability (Figure 3).

Together, these results demonstrate a consistent positive effect of Quercetin and Naringenin on metabolic activity across two independent 2D cellular platforms, fibroblasts (NIH/3 T3) cultured under FGF2-free conditions and bMSCs cultured under reduced-serum conditions. In both systems, extremely low concentrations of the polyphenol salts produced the strongest enhancement in metabolic activity (Figures 2, 3).

### Potassium Naringenin and Sodium Quercetin enhance growth of bESC aggregates in the absence of FGF2

Having established that Quercetin and Naringenin salts improve metabolic activity in 2D culture systems under either FGF2-free (NIH/3 T3, Figure 2) or reduced-serum (bMSC, Figure 3) conditions, we next examined whether these effects extend to three-dimensional cultures of bESCs. For this purpose, we employed a defined suspension aggregation system that supports bESC growth and differentiation under serum-free. This platform provides a more relevant model for cultivated meat production, allowing the evaluation of growth-promoting factors without the confounding effects of serum-derived components. Using this system, we tested whether Potassium Naringenin (K-Ng) and Sodium Quercetin (Na-Q) could promote the growth of bESC aggregates in the complete absence of FGF2.

**Figure 4:**
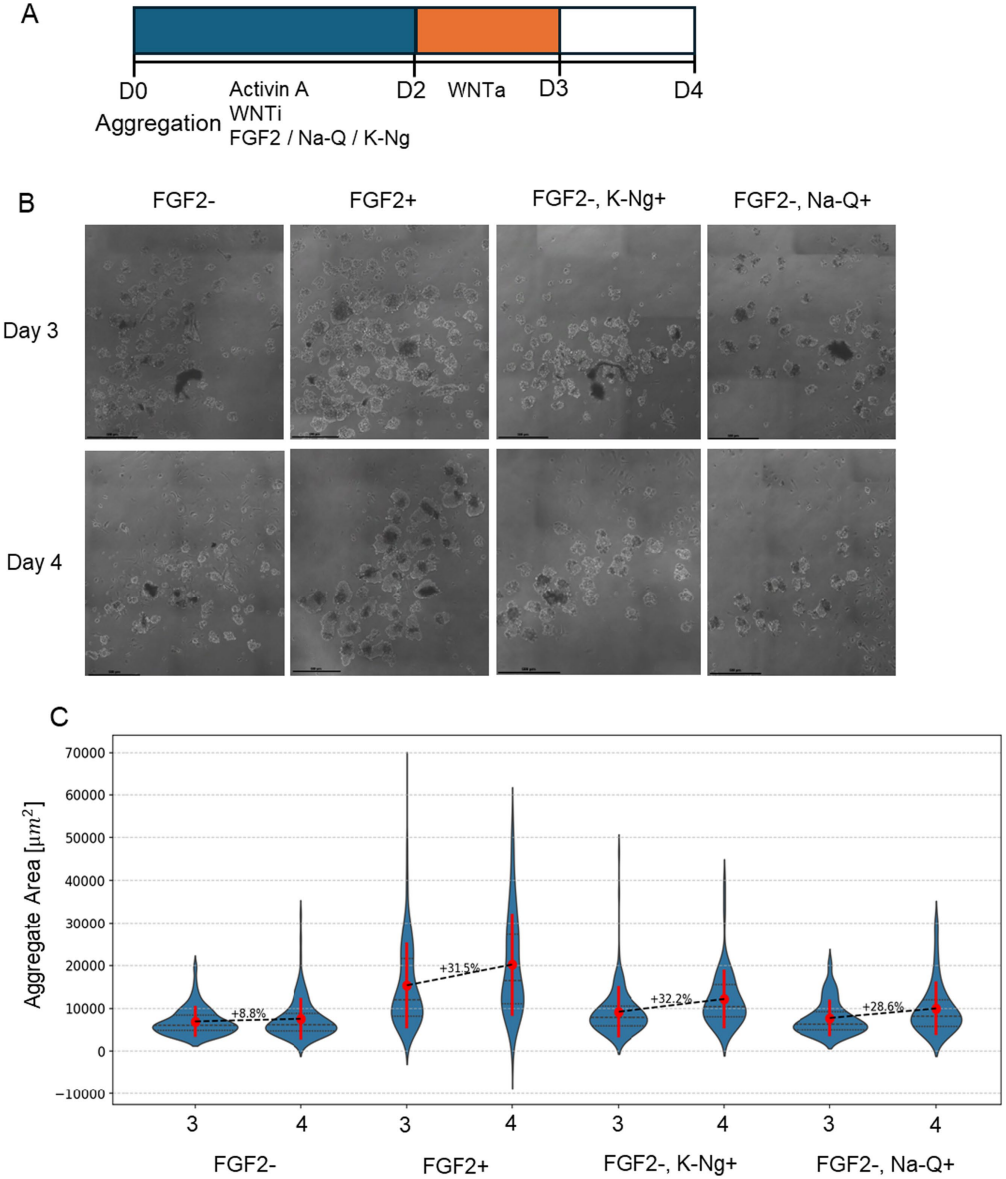
K-Ng and Na-Q improve bESC-based aggregate growth in suspension in the absence of FGF2. **(A)** Schematic representation of the experimental timeline. **(B)** Representative brightfield images of cell aggregates under different conditions. Scale bar = 100um. **(C)** Mean aggregate area (μm^2^) measured on Day 3 and Day 4 for each condition. Violin plots display the distribution of aggregate sizes (*N* > 50 aggregates for each sample), with red marks representing the mean ± standard deviation. The number above each dashed line indicates the percent increase in mean aggregate area from Day 3 to Day 4. Each condition was performed in two wells (replicates), with each well containing at least 15 aggregates.

We cultured bESC aggregates in suspension, where between 0–48 h the medium is supplemented with one of FGF2, Sodium Quercetin (Na-Q) or Potassium Naringenin (K-Ng), or no supplements. To evaluate the Na-Q and K-Ng effect on growth, we monitored aggregates for size progression from Day 3 to Day 4 post-aggregation, as bESC aggregates typically require 3 days to form and stabilize. Brightfield microscopy revealed a marked difference in aggregate size and morphology across the tested conditions (Figure 4B). Quantification of aggregate size demonstrated that, as expected, FGF2 supplementation significantly promoted growth between Day 3 and Day 4 (*p* < 0.005, Dunn’s post-hoc test; Figure 4C). In the absence of FGF2, aggregate growth was substantially reduced. However, the addition of either Na-Q or K-Ng significantly improved aggregate expansion compared to the no-supplementation control (*p* < 0.01 for both). Despite this improvement, neither compound fully recapitulated the effect of FGF2, as both conditions yielded significantly smaller aggregates than the FGF2-treated group (*p* < 0.001).

A common challenge in suspension cultures is that aggregates tend to fuse over time, potentially confounding growth measurements. Since our goal was to assess the effect of polyphenol salts specifically on proliferation rather than aggregate enlargement due to merging, we performed a complementary analysis to control for this variable. Histograms of the full aggregate population on Day 4 (Supplementary Figure 1) revealed a bimodal distribution under both FGF2 + and FGF2+, Na-Q + conditions, suggesting a subpopulation of large aggregates likely formed through fusion. To isolate the effect of Na-Q on cell proliferation, we excluded these large aggregates and re-analyzed only the smaller-sized population (Supplementary Figure 2). Notably, Na-Q still significantly increased aggregate size within this filtered population, supporting the conclusion that the enhanced proliferation underlies the observed growth effect, rather than merely enhanced organoid fusion.

These findings indicate that Na-Q and K-Ng can partially substitute for FGF2 in supporting early aggregate expansion, suggesting their potential as cost-effective additives for bESC culture in suspension systems.

### Potassium Naringenin and Sodium Quercetin cannot fully replace FGF2 in supporting mesodermal differentiation

**Figure 5:**
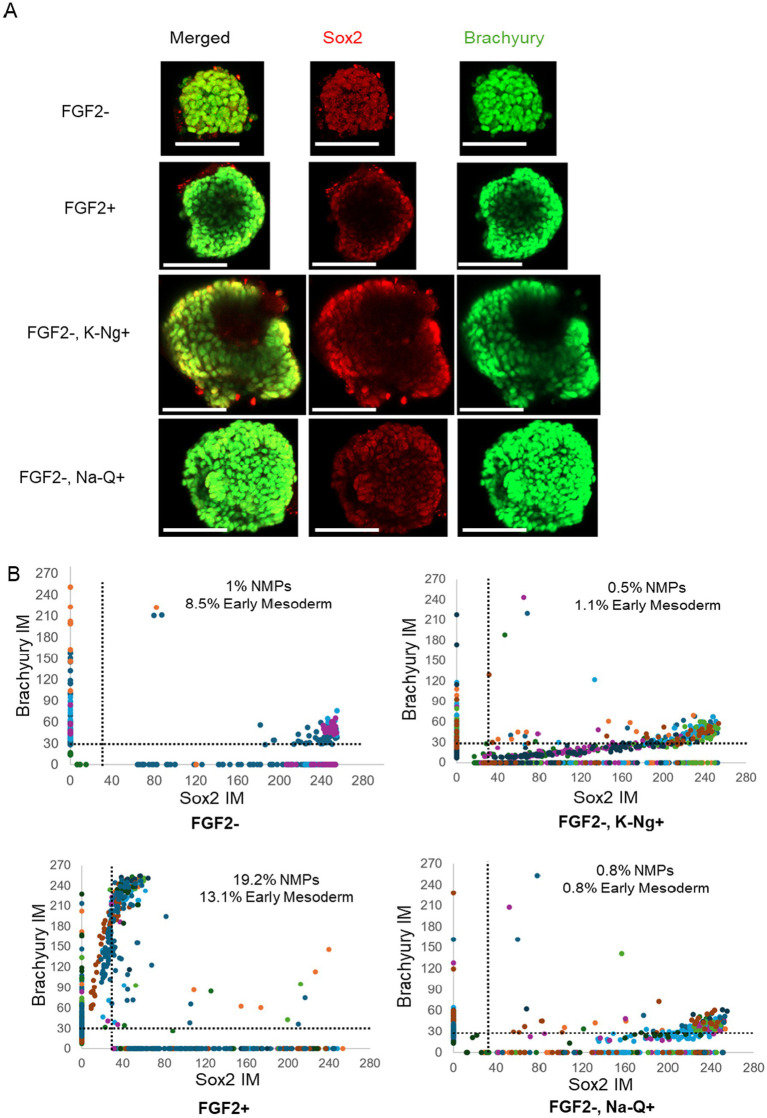
Na-Q and K-Ng do not compensate for FGF2 withdrawal in mesodermal differentiation. **(A)** Immunofluorescence images of a representative aggregate, showing expression of Brachyury (green) and Sox2 (red). Scale bar = 100 μm. **(B)** Single cell fluorescent levels of Brachyury and Sox2 on Day 4 aggregates. Each dot represents a single cell, with different colors indicating different aggregates. Cells expressing Brachyury above threshold designated as early mesoderm, cells expressing Sox2 and Brachyury above the thresholds are designated as NMPs. For single-cell analysis, aggregates were randomly selected from the wells shown in Figure 4: FGF2– K-Ng^+^ (*n* = 8 aggregates), FGF2– Na-Q^+^ (*n* = 10 aggregates), FGF2– (*n* = 5 aggregates), and FGF2^+^ (*n* = 10 aggregates). Each aggregate contained dozens of cells analyzed individually.

We next assessed whether the improved aggregate growth observed with Na-Q or K-Ng supplementation in suspension culture could support mesodermal differentiation in the absence of FGF2. To this end, aggregates were differentiated under serum-free conditions, with or without supplementation of Na-Q or K-Ng and then stained for the early mesodermal marker Brachyury and the pluripotency marker Sox2 (Figure 5A). Single-cell fluorescence analysis was performed to quantify marker expression levels across individual aggregates on Day 4. As expected, FGF2-treated aggregates displayed a robust induction of Brachyury expression, along with the emergence of a distinct population co-expressing Sox2 and Brachyury, characteristic of Neuromesodermal progenitors (NMPs) (Figure 5B, bottom left). In contrast, aggregates cultured without FGF2 showed only minimal Brachyury expression and a lack of defined NMP populations (Figure 5B, top left). Supplementation with Na-Q or K-Ng in the absence of FGF2 did not significantly rescue the differentiation phenotype (Figure 5B, right). Both conditions resulted in limited Brachyury expression and failed to induce the formation of NMP-like populations, indicating that while Na-Q and K-Ng promote aggregate growth, they are insufficient to support mesodermal fate acquisition in the absence of FGF2. These findings suggest that Na-Q or K-Ng cannot fully substitute for FGF2 during early differentiation but may be able to substitute for it during the growth phase.

### Sodium Curcumin enhances bESC aggregate growth and compensates for reduced FGF2 concentration in suspension culture

**Figure 6:**
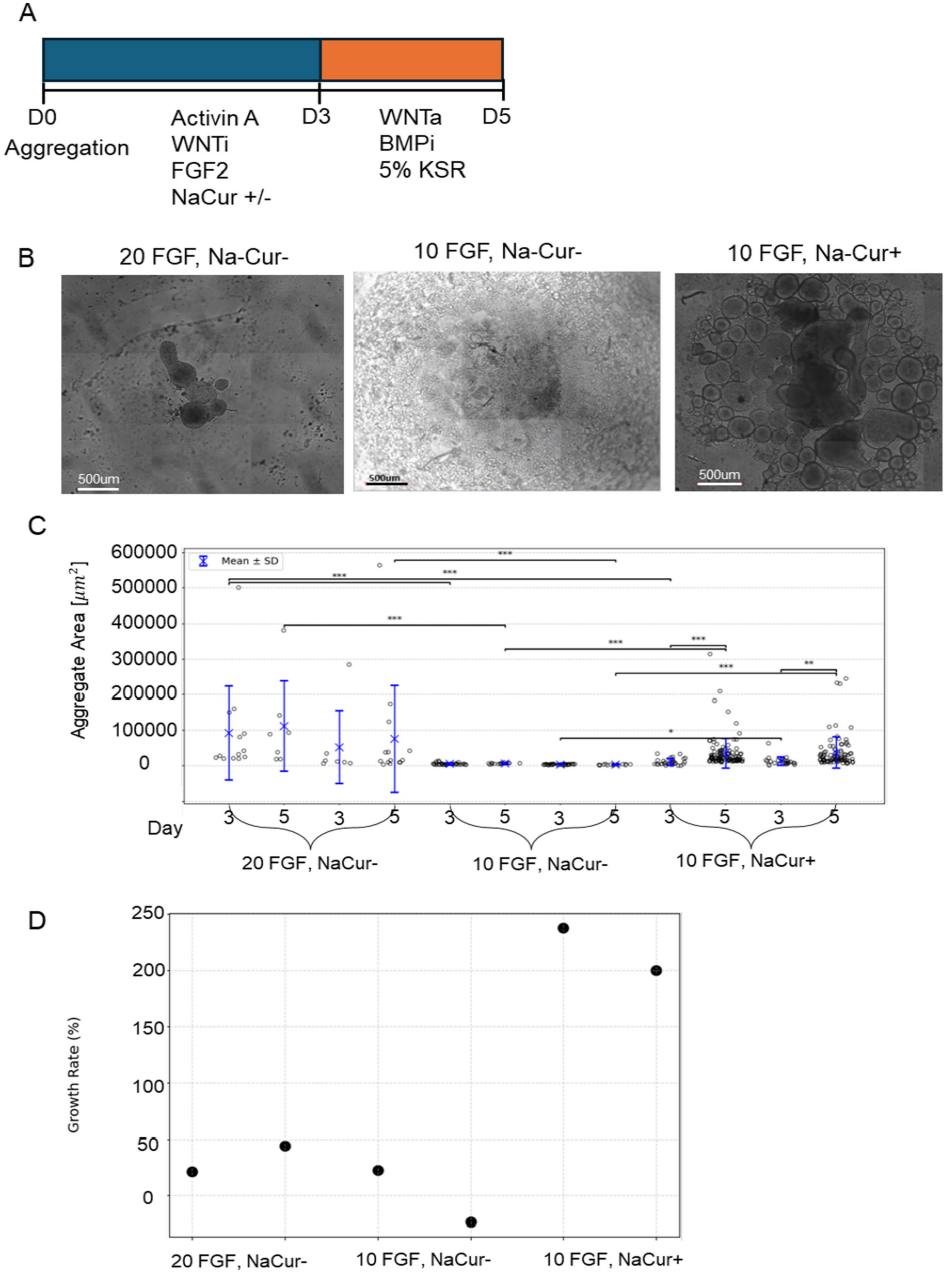
NaCur enhances bESC-based aggregates growth in suspension culture. **(A)** Schematic representation of the differentiation protocol. **(B)** Phase-contrast microscopy images of aggregates on Day 5 of differentiation. Scale bar = 500um. **(C)** Distribution of aggregate size per condition measured on Day 3 and Day 5 in two technical replicates per condition. Individual data points represent single aggregates, with mean values indicated by blue crosses and error bars representing mean ± SD. Statistical comparisons were performed using two-sided *t*-test. Significance was tested between: (1) Day 3 and day 5 within each condition and well. (2) Different FGF2/NaCur conditions measured on the same day and within the same well. Significant differences (*p* < 0.05) are indicated with brackets and asterisks: *p < 0.05, **p < 0.01, ***p < 0.001. **(D)** Growth rate (%) per condition. Growth rate was calculated as the percentage change in aggregate area from Day 3 to Day 5, shown for both technical replicates. Each condition was performed in two wells (replicates), with each well containing at least seven aggregates.

After demonstrating that Na-Q and K-Ng can promote aggregate growth in the absence of FGF2 but cannot support mesodermal differentiation, we next examined whether Curcumin could enhance bESC proliferation and compensate for reduced FGF2 concentration. To this end, we employed a second bESC system based on a different cell line and medium composition, in which FGF2 supplementation is mandatory for cell survival. Before testing Curcumin’s effects in 3D aggregates, we first assessed whether increasing concentrations of NaCur could negatively affect cell viability in a simpler 2D setup. In contrast to Quercetin and Naringenin, elevated NaCur concentrations did not impair bESC proliferation (Supplementary Figure 3), consistent with the NIH/3 T3 results shown in Figure 2. We next evaluated whether NaCur could compensate for the withdrawal or reduction of FGF2 in 2D culture. While NaCur supplementation could not fully rescue cells from complete FGF2 removal, it successfully maintained proliferation when FGF2 concentration was reduced by half (Supplementary Figure 4). Building on these findings, we next tested whether NaCur can also compensate for reduced FGF2 levels in three-dimensional suspended aggregates (Figure 6). We have established a robust 3D aggregate protocol capable of supporting bESC growth under suspension conditions in 24-well plates, generating dozens of aggregates per well (Figure 6A). Importantly, this system enables consistent mesodermal differentiation of the aggregates, a key developmental step toward muscle tissue formation. We tested the effect of NaCur to evaluate whether FGF2 concentration could be reduced without compromising aggregate growth. As initial experiments showed NaCur does not support aggregates growth in complete absent of FGF2, we cultured aggregates in suspension with either 10 ng/mL or 20 ng/mL FGF2, in the presence or absence of 20 nM NaCur, and their area was measured on Days 3 and 5 post aggregation (Figure 6A). Representative images on Day 5 show notable differences in aggregate size across conditions (Figure 6B).

Quantitative analysis revealed that aggregate size increased significantly from Day 3 to Day 5 under all tested conditions, with the most pronounced growth observed in cultures supplemented with both 10 ng/mL FGF2 and NaCur (Figure 6C). While reducing FGF2 concentration from 20 ng/mL to 10 ng/mL alone led to a clear reduction in aggregate size, the addition of NaCur at 10 ng/mL FGF2 not only reduced this effect but significantly enhanced aggregate growth beyond what was achieved with 20 ng/mL FGF2 alone (Figure 6D).

The lower number of data points measured in the 20 ng/mL FGF2 with NaCur condition reflects aggregate fusion rather than reduced aggregate formation or sampling bias. Higher FGF2 levels promote stronger aggregate fusion during the first days of culture, resulting in fewer but larger aggregates per well. The addition of NaCur partially reproduces this effect: while several aggregates fused into larger structures, a subpopulation of smaller aggregates remained, similar in size to those seen in the 10 ng/mL FGF2 condition without NaCur.

### Sodium Curcumin enhances mesodermal differentiation in suspension culture under reduced FGF2 conditions

**Figure 7:**
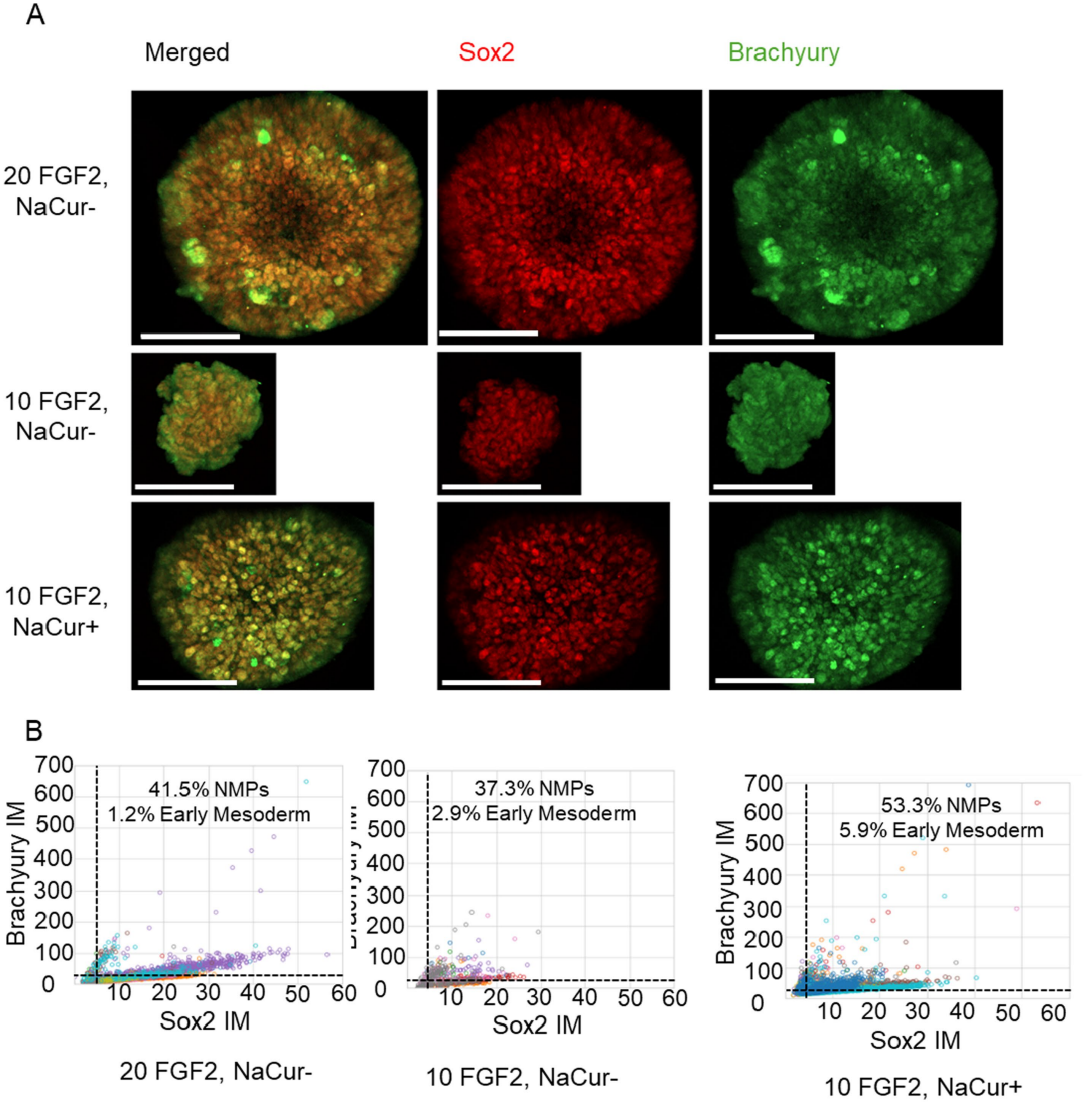
NaCur enhances mesodermal differentiation of bESCs-based aggregates. **(A)** Immunofluorescence images of a representative aggregate, showing expression of Brachyury (green) and Sox2 (red). Scale bar = 100 μm. **(B)** Scatter plots showing single cell fluorescent levels of Brachyury and Sox2 on Day 5 aggregates cultured with 20 ng/mL or 10 ng/mL FGF2, in the presence or absence of 20 nM NaCur. Each dot represents a single cell, with different colors indicating different aggregates. Cells expressing Brachyury above threshold designated as early mesoderm, cells expressing Sox2 and Brachyury above the threshold are designated as NMPs. Aggregates were randomly selected for single-cell analysis from the two wells presented in Figure 4 for each corresponding condition. For the 20 ng/mL FGF2^−^/NaCur^−^ condition, 10 aggregates were analyzed; for the 10 ng/mL FGF2^+^ / NaCur^+^ condition, 11 aggregates were analyzed; and for the 10 ng/mL FGF2^+^ / NaCur^−^ condition, 8 aggregates were analyzed. Each aggregate contained multiple cells analyzed individually for marker expression.

We next investigated whether NaCur supplementation could support mesodermal differentiation of bESC aggregates in suspension under reduced FGF2 concentration. Single-cell immunofluorescence analysis revealed that 20 ng/mL FGF2 strongly induced Brachyury expression and led to the emergence of a co-expressing Sox2 and Brachyury population characteristic of NMPs (Figure 7B). Reducing FGF2 concentration to 10 ng/mL substantially impaired mesodermal differentiation, as indicated by a marked reduction in Brachyury-positive cells. However, the addition of NaCur at 10 ng/mL FGF2 restored Brachyury expression and the NMP population to levels comparable to those observed with 20 ng/mL FGF2. These results demonstrate that NaCur can effectively compensate for reduced FGF2 levels during early mesodermal differentiation in suspension culture.

### Some of the polyphenol salts activate FGFR1 *in vitro*

To explore potential mechanisms underlying the proliferative effect of the polyphenol salts on bESC aggregates, we developed a luciferase-based transcriptional assay to evaluate the activation of human FGFR1. We employed a luciferase reporter assay in COS-7 cells expressing human FGFR1 and a reporter construct. Cells were treated with increasing concentrations of Na-Curcumin, K-Naringenin or Na-Quercetin. We also tested an additional four salts: Na-Naringenin, Na-Apigenin, K-Apigenin and K-Naringin. Luciferase activity was measured as a readout of FGFR1 pathway activation.

**Figure 8:**
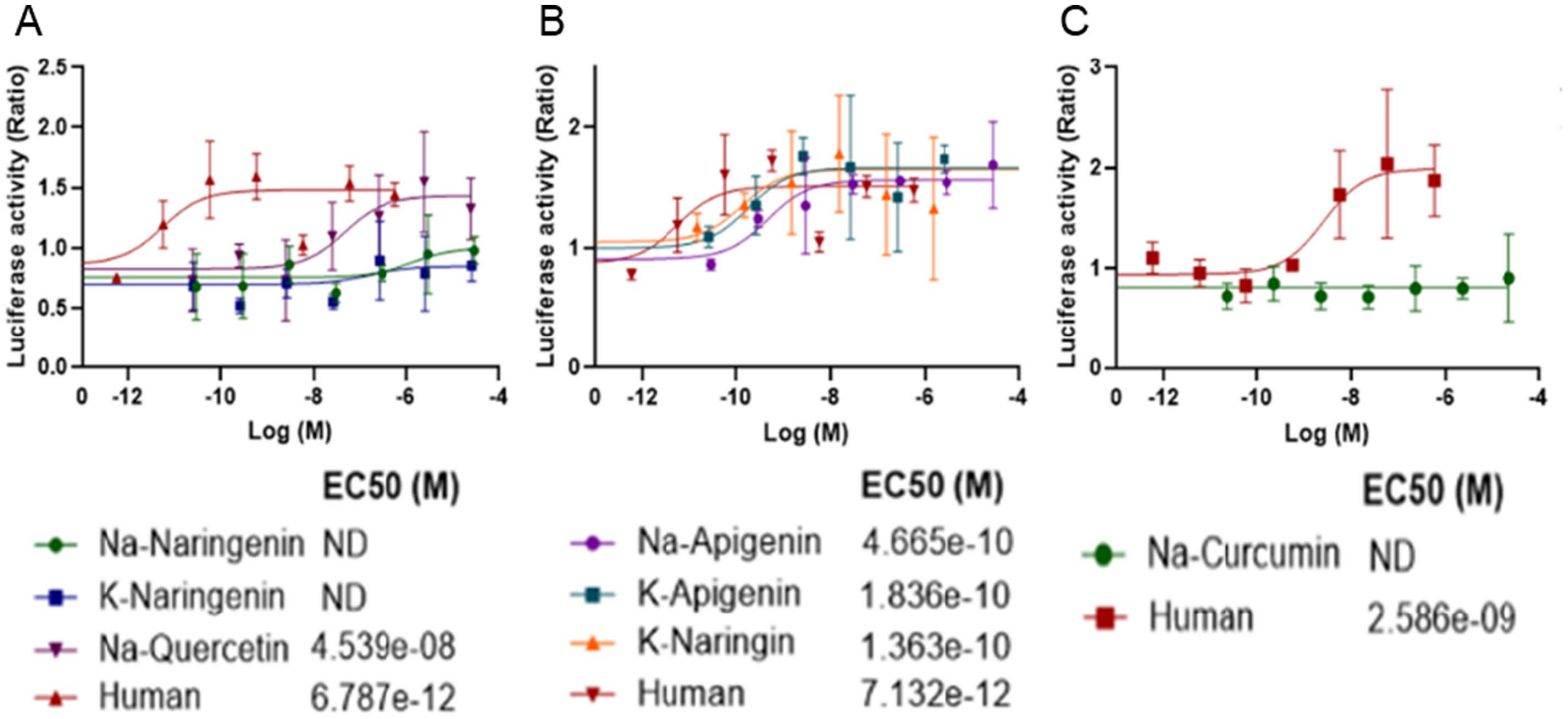
Dose-dependent activation of human FGFR1 by polyphenol salts in a luciferase reporter assay. COS-7 cells were transfected with the human FGFR1, and a reporter construct SRE-LUC and the co-receptor heparan sulphate (10 ng/mL) and treated with increasing concentrations of polyphenol salts **(A)** Na-Naringenin, K-Naringenin and Na-Quercetin. **(B)** Na-Apigenin, K-Apigenin, and K-Naringin **(C)** Na-Curcumin. Human FGF2 served as a positive control. Luciferase activity was measured 6 h after stimulation and is presented as relative luminescence units (RLU) normalized to the untreated control. Each curve represents the mean of at least three independent experiments. Estimated EC₅₀ values indicate the potency of the tested compounds.

K-Naringenin, Na-Quercetin, Na-Apigenin, K-Apigenin and K-Naringin induced a dose-dependent increase in luciferase activity, indicating activation of the human FGFR1 signaling cascade. The dose–response curves revealed high potency with half-maximal effective concentration (EC₅₀) values in the sub-nanomolar range for most compounds (Figure 8). The reference human control compound showed an EC₅₀ at a similar range. These results demonstrate that several polyphenol salts can potently bind and activate the human FGFR1 signaling in vitro, with Na-Quercetin exhibiting the highest potency within the previously tested salts, and K-Apigenin, K-Naringin showing the overall highest potency. The luciferase assay effectively captured the differential activation potential of each compound, providing a quantitative basis for further mechanistic and pharmacological investigation.

These findings suggest that Na-Q may mimic FGF2 activity through direct activation of FGFR1 signaling, providing a mechanistic basis for the enhanced aggregate growth observed in bESC cultures treated with Na-Q. The partial or unstable activation by K-Ng may also contribute to its growth-promoting effects, although to a lesser extent. Together, these data support the hypothesis that certain polyphenol salts can partially substitute for FGF2 during the expansion phase by engaging FGF receptors.

## Discussion

This study demonstrates that polyphenol salts have the potential to partially substitute for FGF2 in NIH/3 T3 mouse fibroblasts (Figure 2) and in two 3D growth models (A-bESC, Figures 4, 5 and R-bESC, Figures 6, 7). In addition, we show that in bMSC, polyphenol salts can compensate for reduced serum concentration, maintaining viability and proliferation under low-FBS conditions. These findings indicate that selected polyphenol salts can either reduce the required concentration of FGF2 or substitute for serum-derived growth support, depending on the cell context.

The NaCur experiments were performed in a system that utilizes native FGF2, a form more likely to meet food-safety and regulatory requirements compared to genetically modified heat-stable FGF2 (HS-FGF2) and supports highly efficient mesodermal differentiation. However, this system includes bovine-derived BSA and does not permit complete FGF2 withdrawal, since aggregate formation is compromised in its absence. In contrast, the K-Ng and Na-Q experiments were conducted in a BSA-free platform in a more defined medium that more closely mirrors future food-grade production standards. While this system required HS-FGF2 and showed slightly reduced efficiency in mesodermal differentiation, it enabled us to assess the effects of polyphenol salts under stringent, serum-free, and scalable conditions on an FGF2-free background. Across these platforms, the three salts exhibited distinct effects on bESC aggregate growth and differentiation.

In the NIH/3 T3 fibroblast assay, metabolic activity measured by MTT was significantly increased by low-nanomolar concentrations of Quercetin, Naringenin and Curcumin salts in the absence of FGF2 (Figure 2). Similarly, in bMSC cultures grown with reduced FBS (3%), supplementation with Naringenin and Quercetin salts sustained cell viability and metabolic activity measured by the Alamar Blue assay (Figure 3). Together, these results establish that polyphenol salts enhance survival and metabolic output in 2D cultures of fibroblast-like and mesenchymal cells under nutrient- or growth-factor-limited conditions. In contrast, metabolic assays were not performed in the bESC system, and proliferation effects there were inferred from aggregate size.

In suspension cultures of R-bESC aggregates, NaCur exhibited a clear positive effect on bESC growth under reduced FGF2 conditions. When combined with a reduced concentration of 10 ng/mL FGF2, NaCur significantly enhanced aggregate growth, even surpassing the size achieved with 20 ng/mL FGF2 alone (Figure 6). This suggests that NaCur can effectively compensate for lower FGF2 levels during the expansion phase. In addition to promoting proliferation, NaCur also supported early mesodermal differentiation, as indicated by Brachyury expression in the presence of low-dose FGF2 (Figure 7). This dual effect highlights the potential of NaCur to function as a cost-effective supplement for improving both growth and lineage commitment in bESC-based systems designed for cultivated meat applications. Supplementary Figure 3 further shows that NaCur modestly enhances proliferation rate of R-bESCs even under fully supplemented FGF2 conditions. Mechanistically, Curcumin is highly lipophilic and can rapidly cross membranes by passive diffusion (17). At low doses, it can activate PI3K/Akt signaling (18) while repressing glucocorticoid-receptor (GR) activity, thereby shifting the GR–STAT3 balance toward STAT3 activation (19). Such dual modulation promotes proliferation and early lineage entry, consistent with NaCur’s ability to compensate for FGF2 reduction. Together with the absence of FGFR1 activation in COS-7 cells (Figure 8C), these findings suggest that NaCur acts downstream or parallel to FGFRs, potentiating PI3K/Akt outputs, promoting adhesion-coupled crosstalk (FAK/ERK → PI3K/Akt), and increasing sensitivity to low-ligand conditions rather than serving as a direct FGF2 agonist.

In A-bESC aggregates, both K-Ng Na-Q demonstrated a clear capacity to enhance bESC aggregate growth in suspension culture under serum-free, FGF2-free conditions (Figure 4). Supplementary Figure 2 indicates that this increase in aggregate size is not due to fusion artifacts, although improved cell viability may also contribute. These findings are particularly encouraging given that the experiments were conducted in a serum-free system, conditions highly relevant to scalable, food-grade cultivated meat production. The COS-7 luciferase assay (Figure 8A) confirmed that Na-Q directly activates human FGFR1 at sub-nanomolar potency, providing a mechanistic explanation for its strong proliferative effect across NIH/3 T3 (Figure 2), bMSC (Figure 3), and bESC (Figure 4) systems.

However, mesodermal differentiation was not achieved under these conditions neither with Na-Q or K-Ng supplementation, as reflected by limited Brachyury expression and absence of a clear NMP-like population in A-bESC aggregates (Figure 5). This dissociation between proliferation and differentiation likely reflects Quercetin’s reported ability to suppress canonical WNT/*β*-catenin signaling by reducing *β*-catenin nuclear accumulation, as reviewed by (20), thereby preventing robust Brachyury induction. Because canonical WNT activation is required for mesodermal entry, Na-Q’s WNT-suppressive activity may constrain differentiation while permitting FGFR-linked proliferation. Importantly, Quercetin has been shown to promote myogenic maturation at later developmental stages, improving MyoD and myogenin expression and myotube formation (21).

Naringenin exhibits hormetic (biphasic) behavior: low concentrations stimulate proliferation and cell survival, whereas higher concentrations can be neutral or inhibitory (22). This pattern aligns with our observations and with previous reports describing naringenin-induced activation of *β*-catenin in regenerative contexts but inhibition in some cancer models (23–26). At later myogenic stages, naringenin has been shown to promote myotube maturation (27), implying that its differentiation effects are stage-dependent and may emerge after mesodermal commitment. It is plausible that different polyphenol salts could be selectively applied to distinct stages of cultivated meat production, with some compounds optimized for expansion and others for lineage-specific differentiation.

Notably, the COS-7 assay (Figure 8) found newer polyphenol salts (K-Naringin, K-Apigenin, An-Apigenin) show more potent activation through the FGFR1 receptor. These salts therefore show potential to serve as FGF2 substitutes for cultivated meat applications as well.

Taken together, these observations support that polyphenol salts exhibit documented biphasic or hormetic dose–response behaviors, in which very low concentrations promote survival and proliferation, while higher concentrations inhibit growth or induce cytotoxicity. This pattern is supported by several studies: Quercetin shows proliferative effects at low doses but inhibition at micromolar levels (28). Low-dose flavonoids reduce oxidative stress and modestly increase proliferation (29), and naringenin and naringin display similar hormetic characteristics (22). Effective concentrations in this study (0.01–20 nM) are several orders of magnitude lower than the micromolar doses typically reported to inhibit proliferation in cancer models, placing our systems on the pro-survival side of the hormetic curve. In NIH/3 T3 and bMSC cells, this low-dose range enhanced metabolic activity (Figures 2, 3), and in bESC aggregates it promoted aggregate growth under FGF2-limited conditions. Thus, low-dose polyphenols appear to act as redox and signaling modulators that sustain PI3K/Akt and STAT3 pathways, maintaining growth and early differentiation capacity under reduced external stimulation.

While our 5-day culture experiments demonstrate that Sodium-Curcumin (NaCur) can enhance bESC aggregate growth and support mesodermal differentiation under reduced FGF2 conditions, we note that long-term culture analysis across multiple passages and metabolic profiling would be necessary to fully validate the use of polyphenol salts in industrial-scale applications. Future studies should assess their effects on genetic stability, differentiation potential, and aggregate viability over extended periods. The same consideration applies to the analysis of mesodermal differentiation under Na-Q and K-Ng supplementation. Based on numerous prior experiments using this bESC aggregate model, we consistently observe robust mesodermal differentiation by Day 4 under FGF2 supplementation. While it remains possible that Na-Q or K-Ng could induce delayed mesodermal differentiation, such a delay would nonetheless represent a limitation in terms of cost-effectiveness when scaling cultured meat production. Therefore, even a temporal shift in marker expression may indicate impaired applicability of these compounds under industrial conditions. Future studies should investigate a broader range of concentrations and extended time courses to fully evaluate the differentiation potential of Na-Q and K-Ng.

## Methods

### Preparation of polyphenol salts

Polyphenol salts were prepared from the reaction of the polyphenols with sodium or potassium hydroxide in ethanol or in water solutions. In a typical synthesis, Quercetin sodium salt was prepared as follows: 50 g of Quercetin (0.1654 mole) dissolved in 2 L of ethanol was mixed with a 250 mL aqueous solution containing 19.85 g of NaOH (0.4962 mole, 3 eq). Upon mixing the solutions, a dark brown color was observed. The reaction mixture was kept overnight on stirring at 500 rpm. The precipitate (the salt) was collected by filtration and washed two times with 500 mL of ethanol and final wash with 200 mL of acetone. The solvent was evaporated to obtain the salts as brown powder. The potassium salt was obtained similarly by replacing the NaOH with KOH.

An alternative method for the salt synthesis was in aqueous media where fine powder of Quercetin was gradually added to a mole equivalent per phenolic OH groups of NaOH or KOH solution in water at room temperature. After stirring overnight, the insoluble residue was isolated by filtration and the filtrate was evaporated to dryness to obtain the salts. The salts were characterized by FTIR, HNMR and elemental analysis. The salt formation was confirmed by a back reaction of the salts in acidic solution to obtain the original polyphenol in good yield.

**Table 1:** 

K-Naringin	
Chemical formula	C_27_H_29_K_3_O_14_
Molar Mass	694.8 g/mol
Appearance	Dark yellow powder
Melting point	> 300 °C
Solubility in water	~ 10 mg/mL at RT
UV λ_max_	286 nm
NMR	1H NMR (500 MHz, DMSO-d6) δ 8.84 (d, J = 15.7 Hz, 1H), 7.45 (d, J = 16.3 Hz, 3H), 6.82 (s, 2H), 5.40 (s, 1H), 5.16 (s, 1H), 5.09 (d, J = 1.5 Hz, 1H), 4.86 (d, J = 7.0 Hz, 1H), 4.60 (s, 4H), 3.85–3.77 (m, 1H), 3.72–3.64 (m, 2H), 3.52 (dd, J = 11.8, 4.2 Hz, 2H), 3.46–3.08 (m, 27H), 1.18 (d, J = 6.2 Hz, 3H).
FTIR	strong carbonyl (C=O) stretching band at 1659 cm^−1^, and characteristic aromatic C=C stretching bands at 1604 cm^−1^ and 1,510 cm^−1^
Na-Quercetin	
Chemical formula	C_15_H_5_Na_5_O_7_
Molar mass	412.1 g/mol
Appearance	Brown Powder
Melting point	> 300 °C
Solubility in water	~ 1 mg/mL at RT
UV λ_max_	322 nm
NMR	^1^H NMR (500 MHz, DMSO-d_6_) *δ* 12.48 (s, 1H), 10.90 (s, 1H), 9.34 (s, 2H), 7.68 (d, J = 2.2 Hz, 1H), 7.55 (dd, J = 8.5, 2.2 Hz, 1H), 6.90 (d, J = 8.5 Hz, 1H), 6.45 (d, J = 2.1 Hz, 1H), 6.21 (d, J = 2.0 Hz, 1H).
FTIR	strong carbonyl (C=O) stretching band at 1659 cm^−1^, and characteristic aromatic C=C stretching bands at 1604 cm^−1^ and 1,510 cm^−1^

### NIH/3 T3 metabolic activity measurement (MTT assay)

NIH/3 T3 fibroblasts were used to assess the effect of polyphenol salts on cell metabolic activity under serum-starvation and FGF2-free conditions. One day prior to the experiment, 1,500–3,000 cells per well were seeded in 96-well flat-bottom tissue culture plates in maintenance medium containing 10% fetal calf serum (FCS) (F9665, Merck).

On the first day of the experiment, cells were washed once with PBS and the medium was replaced with starvation medium, in which serum concentration was reduced from 10 to 1% FCS. Test compounds were then added according to the experimental condition: 5 ng/mL FGF2 (100-18B-100, Peprotech Asia) as a positive control, or polyphenol salts (Li-, Na-, or K-salts of Quercetin, Curcumin, or Naringenin) at the indicated concentrations (5 nM, 0.1 nM, or 0.01 nM). Cells were cultured for five days at 37 °C and 5% CO₂, without medium replacement.

On Day 5, metabolic activity was assessed by MTT assay. Briefly, MTT solution (5 mg/mL) was added to the wells to obtain a final concentration of 0.5 mg/mL. Cells were incubated for additional two hours. The media was the completely removed and cell lysis was obtained with 50 ul DMSO. After aggressive shaking for a few minutes, the absorption was read using a plate reader, at 540 nm.

Each experimental condition was tested in 5–6 technical replicates and repeated twice. Background absorbance from blank wells was subtracted prior to analysis. Data were normalized to the negative control (FGF2-). Statistical significance was determined using single tail T Test. Significance levels are indicated as *p* < 0.05, *p < 0.01, and **p < 0.001.

### AD-bMSC cell viability assay

AD-bMSCs were generated and maintained as previously described (34). To evaluate the effect of polyphenol salts on bMSC survival under low-serum conditions, a dose–response assay was performed in 96-well plates. Na-Ng, K-Ng, Na-Q, and K-Q salts were freshly reconstituted in growth medium immediately before use to reach final concentrations ranging from 5 nM to 0.01 nM.

Cells were seeded at a density of 2,000 cells per well in 96-well tissue-culture plates (Lifegene, Israel) in six technical replicates per condition. Following seeding, cells were allowed to attach overnight in growth medium containing 15% FBS at 37 °C and 5% CO₂ to ensure uniform initial density. The next day, the medium was replaced with treatment medium consisting of 3% FBS + 1% ITS % ITS-Plus Media Supplement (R&D systems), with or without the indicated polyphenol salt. A parallel 15% FBS condition served as a full-serum control. Cells were cultured for 5 days, with one medium refreshment on day 3.

Cell metabolic activity, used as a proxy for viability and proliferation, was assessed on day 5 using the Resazurin (Alamar Blue) colorimetric assay (Merck, United States). Briefly, culture medium was replaced with 100 μL of 15% FBS medium supplemented with 10 μL of Alamar Blue reagent to achieve a final Resazurin concentration of 44 μM. After 3 h of incubation at 37 °C and 5% CO₂, absorbance was measured using a BioTek Synergy HTX multimode plate reader at 570 nm (reference 600 nm).

Absorbance values were normalized to the negative control (3% FBS + 0 nM), and data are presented as normalized absorbance (540/590 nm). Each individual well is plotted as an empty symbol (○ rep a, □ rep b, △ rep c), with blue crosses representing mean ± SD of replicates. One-sided t-tests were performed per replicate against the indicated control. Statistical significance is denoted as *p* < 0.05 (), *p* < 0.01 (), *p* < 0.001 (); brackets summarize per-rep significance, shown only where at least one replicate reached significance.

### bESC cell culture and maintenance

Two bovine embryonic stem cell (bESC) lines were used in this study. The first, referred to as A-bESC, was obtained from the Alberio laboratory and was originally established as described by Kinoshita et al. (15). The second, referred to as R-bESC, was provided by the Ross laboratory and was established as detailed by Bogliotti et al. (14).

To maintain pluripotency, bESCs were cultured in 2D on mitotically inactivated mouse embryonic fibroblasts (iMEFs) seeded on tissue culture-treated plastic plates coated with 0.2% gelatin. Cultures were maintained at 37 °C and 5% CO₂. The basal medium was N2B27 supplemented with 20 ng/mL Activin A (338-AC, R&D Systems), FGF2, and a Wnt inhibitor, either 2.5 μM IWR-1 (I0161, Merck) for R-bESC or 2 μM XAV939 (X3004, Merck) for A-bESC. Additionally, the R-bESC medium included 1% BSA (9048-46-8, MPBIO) and was supplemented with 20 ng/mL native (non-heat-stable) FGF2 (100-18B, ThermoFisher), whereas the A-bESC, supplemented with 12.5 ng/mL heat-stable FGF2 (HS-FGF2, 100-18BHS, ThermoFisher). Cells were passaged upon reaching approximately 80% confluence using either TrypLE Express (12,604,013, ThermoFisher) or Accutase.

### Aggregate formation and differentiation in suspension culture

To initiate aggregate formation, bESCs were dissociated into single cells using TrypLE Express or Accutase, as done for routine passaging. Cells were seeded into uncoated, feeder-free tissue culture plates and maintained in suspension on an orbital shaker at 125RPM for R-bESC and 150RPM for A-bESC. Aggregation was carried out in the pluripotency maintenance medium (N2B27 supplemented with Activin A, Wnt inhibitor, and FGF2 or polyphenol salts, depending on the condition). The seeding density was 120,000 cells/mL for the R-bESC line and 65,000 cells/mL for the A-bESC line.

Experimental conditions included either complete FGF2 withdrawal, reduced FGF2 concentration (10 ng/mL non-heat-stable FGF2), or FGF2 replacement with polyphenol salts. For the K-Ng and Na-Q experiments, 1 nM of the respective salt was added without FGF2. A concentration of 1 nM was chosen for initial testing of Na-Q and K-Ng based on preliminary experiments showing no toxicity and FGF receptors biding. The selected concentration does not exclude the possibility that higher doses of Na-Q or K-Ng may affect differentiation outcomes. Future dose–response experiments will be required to evaluate this possibility. For NaCur testing, 10 ng/mL non-heat-stable FGF2 was combined with 20 nM NaCur. Positive controls included either 20 ng/mL non-heat-stable FGF2 or 12.5 ng/mL heat-stable FGF2.

From the end of the aggregation phase onward, cell line-specific differentiation protocols were applied:

R-bESC line: Aggregation continued for 3 days (D0–D3). On Day 3, medium was replaced with differentiation medium consisting of N2B27 supplemented with 10 μM CHIR99021 (WNT agonist), 0.5 μM LDN193189 (BMP inhibitor, SML0559, Merck), and 5% KnockOut Serum Replacement (KSR), and maintained until Day 5.

A-bESC line: Aggregation lasted for 2 days (D0–D2). On Day 2, cells were pulsed with 10 μM CHIR99021 in N2B27 for 24 h. On Day 3, the medium was replaced with plain N2B27 (no supplements), which was maintained until Day 4.

Both lines remained in suspension under continuous shaking throughout the entire culture period.

NaCur experiments on R-bESC aggregate growth and differentiation were conducted in two independent experimental runs, each including two technical replicates (individual wells). Each well contained multiple aggregates analyzed separately for quantitative measurements.

A-bESC experiments assessing the effects of K-Ng and Na-Q on aggregate growth and differentiation were conducted in a single experiment including two technical replicates (individual wells). Each well contained multiple aggregates analyzed separately for quantitative measurements.

### Aggregate size analysis

Live aggregates were imaged on Day 3 and Day 4 or Day 5 using an epifluorescence microscope in brightfield mode. To ensure complete sampling, the entire well was imaged using a tiled acquisition approach with automated stitching, allowing all aggregates within each well to be captured and analyzed. Aggregate size was quantified using FIJI (ImageJ) by manually outlining the perimeter of each aggregate and measuring the projected 2D area in square microns (μm^2^).

For statistical analysis of aggregate area distributions, Kruskal-Wallis tests were performed followed by Dunn’s post-hoc test with Bonferroni correction.

Aggregate size was used as a proxy for growth, based on common practice in 3D stem cell culture systems, where changes in size often correlate with changes in total cell number. In our system, single-cell immunostaining and imaging of multiple aggregates per condition did not reveal prominent lumen formation or internal cavities, supporting the interpretation that increased size reflects higher cell number.

### Immunofluorescence analysis

Aggregates from each experimental condition were pooled and fixed in 4% paraformaldehyde, either overnight at 4 °C or for 1 h at room temperature. Fixed aggregates were then blocked and permeabilized overnight at 4 °C in PBS containing 7.5% fetal bovine serum and 0.2% Triton X-100.

Primary antibody incubation was performed for 2–3 days at 4 °C in blocking buffer. The following primary antibodies were used: anti-Brachyury (AF2085, R&D Systems) at 1:10 dilution, and anti-Sox2 (149,881,182, Thermo Fisher) at 1:200 dilution. After incubation, aggregates were washed twice in blocking buffer and incubated again in blocking solution overnight. Secondary antibodies were then applied overnight at 4 °C at a dilution of 1:500.

Following staining, aggregates were washed and transferred to glass-bottom plates, embedded in 0.5% low-melting-point agarose, and imaged using a confocal microscope. Z-stacks were acquired in two fluorescence channels.

Image analysis was performed using Imaris. Stained cells were segmented in 3D to extract their Euclidean (x, y, z) coordinates and mean fluorescence intensity in each channel. This data was used to assign marker expression levels across aggregates.

### Statistical analysis

Statistical analyses were performed using custom Python scripts based on SciPy and NumPy libraries. For all tests, *p*-values < 0.05 were considered statistically significant, and significance levels are indicated in the figure legends as *p* < 0.05 (), *p* < 0.01 (), and *p* < 0.001 ().

For NIH/3 T3 metabolic activity assays (Figure 2), one-sided unpaired t-tests (alternative hypothesis: greater) were performed to compare each treatment group to the negative control (FGF2–).

For bMSC survival assays (Figure 3), one-sided Welch’s t-tests (unequal variances, alternative hypothesis: greater) were applied to compare each polyphenol treatment with the negative control.

For bESC aggregate growth assays with K-Ng and Na-Q (Figure 4) and with NaCur (Figure 6), nonparametric Kruskal–Wallis tests were performed followed by Dunn’s *post hoc* tests with Bonferroni correction for multiple comparisons.

All analyses were based on independent wells as biological replicates, as detailed in the figure legends.

### Transactivation of human FGF1 receptor with Curcumin salts

We evaluated the efficacy of Curcumin-derived salts in activating human FGFR1 and compared their performance to that of the native ligand, human FGF2. This comparison allowed us to assess whether these compounds can mimic or surpass the biological activity of FGF2 in triggering FGFR1-mediated signaling pathways.

FGFs exert their pleiotropic effects by binding and activating high-affinity tyrosine kinase receptors that are coded by four genes (FGFR1-4), when FGF2 signals through the FGFR1 (30). Specific ligands typically initiate signal transduction by forming a ternary complex at the cell surface, involving the ligand, its receptor, and a co-receptor such as heparan sulfate or Klotho proteins. This complex stabilizes receptor activation and triggers downstream signaling pathways. As a paracrine FGF, the co-receptor of FGF2 is heparan sulfate (31).

The entire coding sequence of the human FGFR1 (accession no. AAH15035.1) was inserted into pcDNA3.1 (Invitrogen) and verified by sequencing. The procedures for transient transfection of the COS-7 cells and receptor stimulation have been described previously (32, 33). In brief, COS-7 cells were cotransfected with a luciferase reporter plasmid (SRE-luc; 3 μg) and the hFGFR1 (3 μg). As a control treatment, the receptors were transfected without reporter plasmid (data not shown). After 48 h, the transfected cells were exposed to increasing concentrations of the Curcumin salts and human FGF2 as a positive control. Six hours after stimulation, the cells were analyzed using the GloMax multidetection system (Promega).

## Data Availability

The raw data supporting the conclusions of this article will be made available by the authors, without undue reservation.

## Supplementary material

SUPPLEMENTARY FIGURE 1 Size distribution of the full aggregate population reveals potential fusion events. Histograms showing the distribution of aggregate areas measured on day 4 under four conditions: FGF2+, FGF2−, FGF2−, K-Ng+, and FGF2-, Na-Q+. In the FGF2+ and FGF2−, Na-Q+ conditions.

SUPPLEMENTARY FIGURE 2 Na-Q promotes proliferation of bESC aggregates independent of fusion artifacts. Histograms showing the size distribution of only the smaller aggregate population (large aggregates excluded). The exclusion of large, likely fused aggregates allows for a more accurate assessment of proliferation-driven growth. Even within this filtered population, aggregates cultured with Na-Q in the absence of FGF2 remain significantly larger than those without any supplementation, confirming that Na-Q enhances cell proliferation rather than promoting aggregate fusion.

SUPPLEMENTARY FIGURE 3 NaCur modestly enhances proliferation rate of R-bESCs under standard FGF2-supplemented conditions. R-bESCs were cultured for a total of 10 days and passaged three times (P1–P3) under pluripotency-maintaining conditions containing 20 ng/mL FGF2, with or without the addition of NaCur at the indicated concentrations. Cells were counted using a hemocytometer, and viability was determined by Alamar Blue staining. **(A)** Percentage viability per passage. NaCur supplementation at concentrations up to 20 nM did not affect cell viability. **(B)** Growth ratio calculated as (final cell number / initial cell number) / number of days per passage. Addition of NaCur at 10–20 nM slightly increased proliferation rate relative to the 20 ng/mL FGF2 control, suggesting that NaCur may enhance cell growth even under fully supplemented conditions.

SUPPLEMENTARY FIGURE 4 R-bESCs were cultured for a total of 10 days, during which they were passaged three times (P1–P3), under pluripotency-maintaining conditions containing different concentrations of FGF2 (20 ng/mL, 10 ng/mL, or none) with or without 20 nM NaCur. **(A)** Phase-contrast images show that in the absence of FGF2, cells failed to survive beyond P1. Whereas supplementation with 20 nM NaCur enabled robust proliferation under reduced (10 ng/mL) FGF2 conditions. **(B)** Quantification of growth ratio (cells/day) based on Alamar Blue and hemocytometer counts (bottom) indicates that NaCur supplementation-maintained proliferation rates comparable to or exceeding those observed with 20 ng/mL FGF2 alone. Data represent mean ± SD of two technical replicates per condition. Significant differences (*p* < 0.05) are indicated with brackets and asterisks: **p* < 0.05, ***p* < 0.01, ****p* < 0.001.
