# Supplementary figures and images for: Toward FGF2 reduction in cultured meat media: polyphenol salts enhance growth and differentiation of bESC aggregates
Source: Front Nutr. 2025 Dec 3;12:1669909. doi: 10.3389/fnut.2025.1669909 (PMC12709626; doi:10.3389/fnut.2025.1669909)

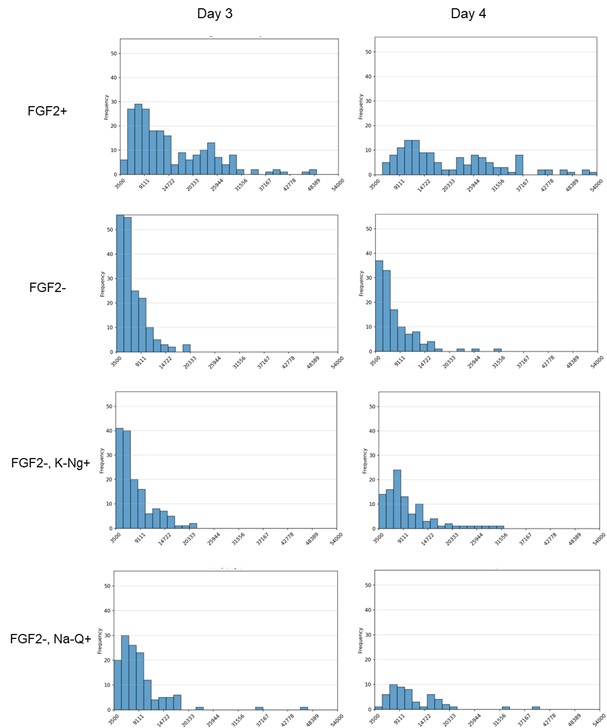

Supplement: SUPPLEMENTARY FIGURE 1 — Size distribution of the full aggregate population reveals potential fusion events. Histograms showing the distribution of aggregate areas measured on day 4 under four conditions: FGF2+, FGF2−, FGF2−, K-Ng+, and FGF2-, Na-Q+. In the FGF2+ and FGF2−, Na-Q+ conditions. [file Image_1.JPEG]

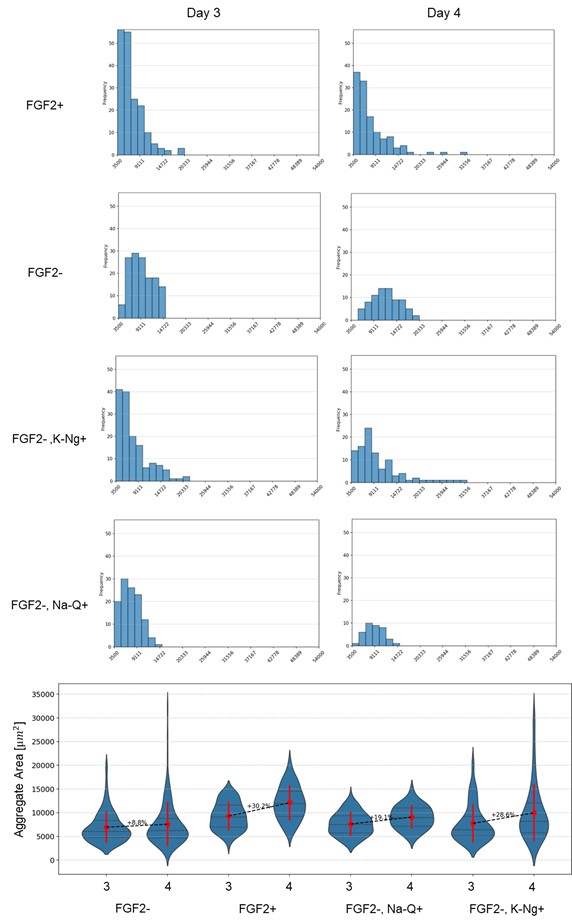

Supplement: SUPPLEMENTARY FIGURE 2 — Na-Q promotes proliferation of bESC aggregates independent of fusion artifacts. Histograms showing the size distribution of only the smaller aggregate population (large aggregates excluded). The exclusion of large, likely fused aggregates allows for a more accurate assessment of proliferation-driven growth. Even within this filtered population, aggregates cultured with Na-Q in the absence of FGF2 remain significantly larger than those without any supplementation, confirming that Na-Q enhances cell proliferation rather than promoting aggregate fusion. [file Image_2.JPEG]

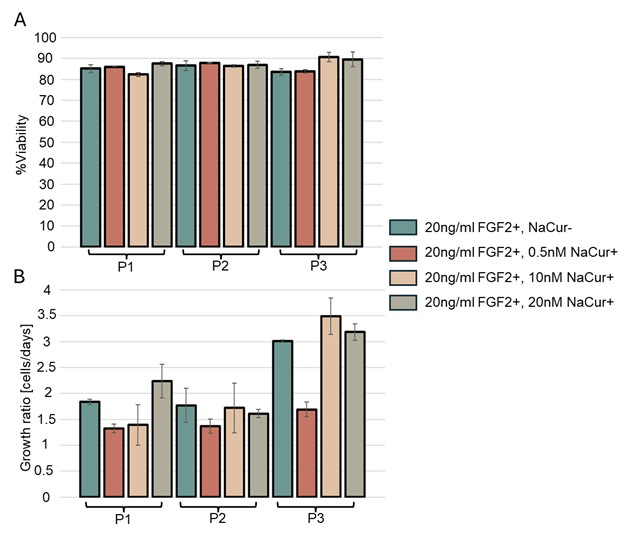

Supplement: SUPPLEMENTARY FIGURE 3 — NaCur modestly enhances proliferation rate of R-bESCs under standard FGF2-supplemented conditions. R-bESCs were cultured for a total of 10 days and passaged three times (P1–P3) under pluripotency-maintaining conditions containing 20 ng/mL FGF2, with or without the addition of NaCur at the indicated concentrations. Cells were counted using a hemocytometer, and viability was determined by Alamar Blue staining. (A) Percentage viability per passage. NaCur supplementation at concentrations up to 20 nM did not affect cell viability. (B) Growth ratio calculated as (final cell number / initial cell number) / number of days per passage. Addition of NaCur at 10–20 nM slightly increased proliferation rate relative to the 20 ng/mL FGF2 control, suggesting that NaCur may enhance cell growth even under fully supplemented conditions. [file Image_3.JPEG]

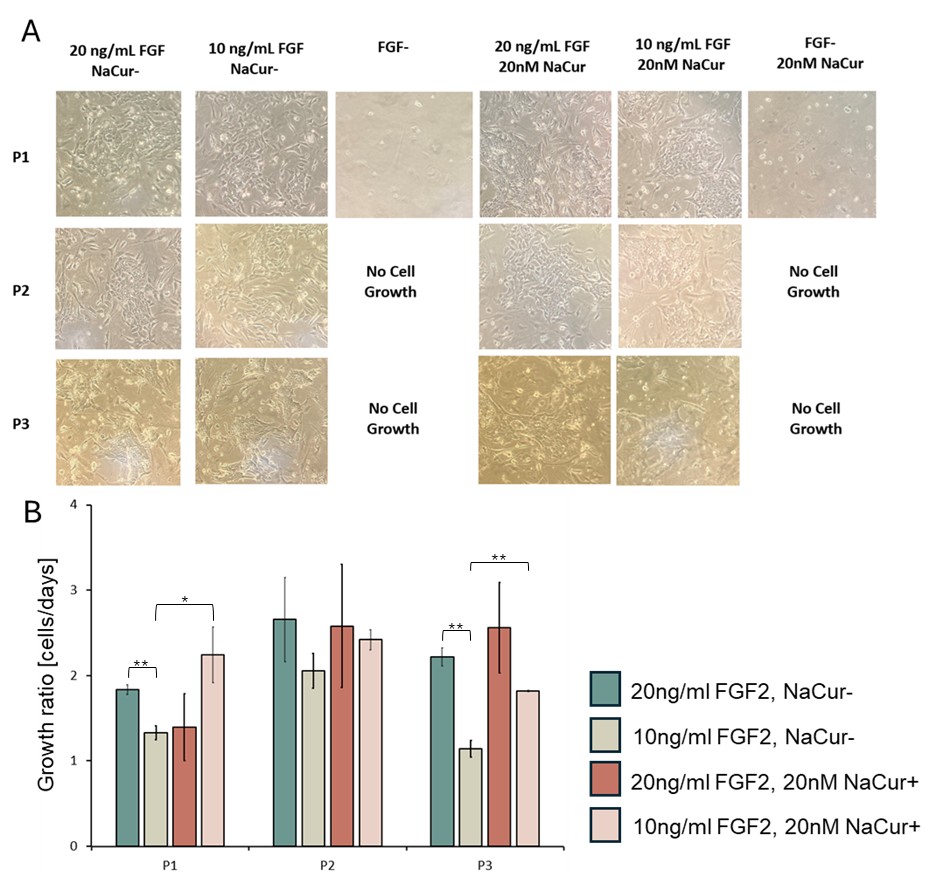

Supplement: SUPPLEMENTARY FIGURE 4 — R-bESCs were cultured for a total of 10 days, during which they were passaged three times (P1–P3), under pluripotency-maintaining conditions containing different concentrations of FGF2 (20 ng/mL, 10 ng/mL, or none) with or without 20 nM NaCur. (A) Phase-contrast images show that in the absence of FGF2, cells failed to survive beyond P1. Whereas supplementation with 20 nM NaCur enabled robust proliferation under reduced (10 ng/mL) FGF2 conditions. (B) Quantification of growth ratio (cells/day) based on Alamar Blue and hemocytometer counts (bottom) indicates that NaCur supplementation-maintained proliferation rates comparable to or exceeding those observed with 20 ng/mL FGF2 alone. Data represent mean ± SD of two technical replicates per condition. Significant differences (p < 0.05) are indicated with brackets and asterisks: *p < 0.05, **p < 0.01, ***p < 0.001. [file Image_4.JPEG]
